# Employing Endogenous NSCs to Promote Recovery of Spinal Cord Injury

**DOI:** 10.1155/2019/1958631

**Published:** 2019-05-05

**Authors:** Sumei Liu, Zhiguo Chen

**Affiliations:** ^1^Cell Therapy Center, Beijing Institute of Geriatrics, Xuanwu Hospital, Capital Medical University, Beijing 100053, China; ^2^Key Laboratory of Neurodegenerative Diseases, Ministry of Education, Beijing 100053, China; ^3^Center of Neural Injury and Repair, Beijing Institute for Brain Disorders, Beijing 100069, China; ^4^Center of Parkinson's Disease, Beijing Institute for Brain Disorders, Beijing 100069, China

## Abstract

Endogenous neural stem cells (NSCs) exist in the central canal of mammalian spinal cords. Under normal conditions, these NSCs remain quiescent and express FoxJ1. After spinal cord injury (SCI), the endogenous NSCs of a heterogeneous nature are activated and proliferate and migrate towards the lesion site and mainly differentiate into astrocytes to repair the injured tissue. *In vitro*, spinal cord NSCs are multipotent and can differentiate into neurons, astrocytes, and oligodendrocytes. The altered microenvironments after SCI play key roles on the fate determination of activated NSCs, especially on the neuronal specification potential. Studies show that the activated spinal cord NSCs can generate interneurons when transplanted into the adult hippocampus. In addition, the spinal cord NSCs exhibit low immunogenicity in a transplantation context, thus implicating a promising therapeutic potential on SCI recovery. Here, we summarize the characteristics of spinal cord NSCs, especially their properties after injury. With a better understanding of endogenous NSCs under normal and SCI conditions, we may be able to employ endogenous NSCs for SCI repair in the future.

## 1. Introduction

Neural stem cells (NSCs) exist mainly in two regions in the adult central nervous system (CNS): brain and spinal cord [1–6]. NSCs remain quiescent under normal physiological conditions and can be activated under certain conditions such as CNS injury [7]. The activated NSCs can self-renew to maintain stem cell pool size and differentiate into neural cells for tissue repair. In this review, we will focus on the properties and behavior of endogenous spinal cord NSCs in normal situations and SCI.

## 2. Endogenous Spinal Cord NSCs

The identity of endogenous spinal cord NSCs has been debated in the past years. Astrocytes, oligodendrocyte progenitors, and ependymal cells have all been suggested as spinal cord stem cells previously by different groups [8–10]. Cortical astrocytes gain some NSC properties and assume a proliferative status after brain injury, but these cells cannot give rise to cell types other than astrocytes [11]. Some studies have suggested that oligodendrocyte progenitors can differentiate into astrocytic lineage in addition to oligodendrocytes, but recent studies failed to prove this and these cells seem to have a restricted potential for oligodendrocyte lineage only [12–14]. The only cell type that has been confirmed to be multipotent and retains a neurosphere-forming and passaging ability *in vitro* is ependymal cells [8]. What is noteworthy is that some parenchymal progenitors in regions other than the ependymal zone can proliferate after SCI and give rise to neurons and glia *in vitro*, but these cells can hardly be expanded *in vitro* (fewer than 2-3 passages) [15, 16].

### 2.1. Spinal Cord NSCs Lining the Central Canal

Ependymal cells lining the central canal are referred as spinal cord NSC niche. In the mouse spinal cord, ependymal cells originate at mid embryonic stages (E15.5) and completely surround the central canal by birth (P0) [17]. Based on studies on FoxJ1-CreER transgenic mice, the ependymal cells can be categorized into 3 basic types according to cell morphology rather than function: cuboidal ependymal cells, tanycytes, and radial ependymal cells [10]. Cuboidal ependymal cells are the most abundant multiciliated cells, and the radial ependymal cells are the less numerous type [18]. In adult macaque, there are 3 subtypes according to the number of cilia: uniciliated, biciliated, and multiciliated, and the first two subtypes give rise to new ependymal cells [19]. In addition, another cell type, cerebrospinal fluid- (CSF-) contacting neurons (CSF-CNs), exists in lower vertebrates, such as fish and amphibians but not in higher mammals such as dogs and cats [20, 21]. NSCs extend an apical protrusion in the ependymal zone to contact with CSF; the flow of CSF can be sensed through a transmembrane sodium channel which can regulate the proliferation of these cells by activating Erk cascade [22].

### 2.2. Ependymal Cells Are Heterogeneous

Studies show that ependymal cells are heterogeneous and express radial glia cell markers such as RC1 and BLBP and NSC markers such as CD15 (also known as Lewis X antigen or SSEA-1), GFAP, PSA-NCAM, Musashi1, CD133/prominin-1, Sox2, Sox3, and Sox9, as well as vimentin and nestin [10, 23–26]. Nestin is expressed in dorsal and ventral poles of ependymal cells and CD15 and BLBP in dorsal regions [24]. In adult mouse spinal cord, the numbers of nestin-containing cells (NCCs) are the greatest in cervical vertebrae 1–7, the second in thoracic 1–12, and the smallest in lumbar 1–5, and no significant difference exists in the left vs. right side [27].

## 3. Response of Endogenous Spinal Cord NSCs after Injury

Following injury, endogenous spinal cord NSCs go through 3 steps: activation, migration, and differentiation.

### 3.1. Spinal Cord NSC Activation

**Figure 1:**
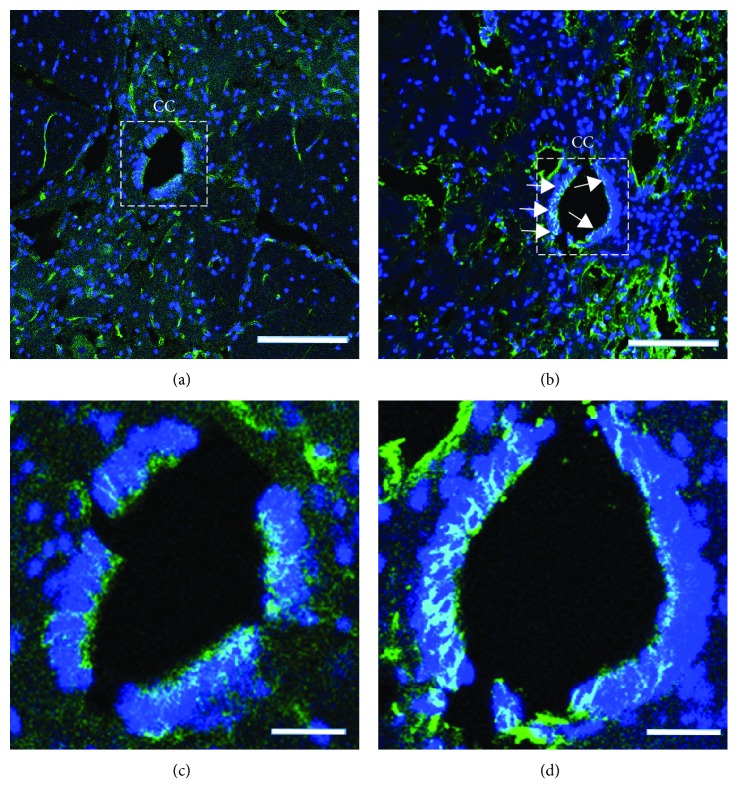
Transverse sections show an elevated expression of nestin in the rat central canal (CC). (a) Intact spinal cord. (b) 3 days after injury. (c and d) Magnified CC of the insets in (a) and (b), respectively. Nestin, green; DAPI, blue. Arrows show the Nestin^+^ cells. Scale bars: (a and b): 200 *μ*m; (c and d): 50 *μ*m.

Studies have shown that spinal cord injury induces activation of spinal cord NSCs which otherwise would remain in a quiescent state or assume very slow proliferation under normal conditions [7, 28–30]. Ependymal cells, astrocytes, and NG2^+^ oligodendrocytes are all stimulated to divide after injury [9, 31, 32]. Spheres derived from injured spinal cords grow 3-4 times faster and larger than those from intact spinal cords [8]. Some ependymal cell markers such as Sox2, Sox3, and FoxJ1 are downregulated when the progeny cells leave the central canal and contribute to glial scar formation [10]. Another cell population called radial glia that express vimentin, BLBP, and nestin increase the expression of these markers after injury [33, 34]. Reports show that SCI increases the number of Nestin^+^/Sox2^+^ cells in the spinal cord, especially in the central canal in rats [35–37]. The expression of nestin, which is a marker of NSCs, is upregulated in the central canal after acupuncture injury with a 25 G needle (Figure 1).

Changes of microenvironment, such as an increased level of certain soluble factors, a hypoxic condition due to vascular destruction, and immune responses, may contribute to NSC activation after CNS injury [38–43]. In human spinal cords, multipotent NSCs have been isolated and studied *in vitro*, and the number of Nestin^+^/GFAP^+^ cells is increased after traumatic injury as examined by histopathological analysis of the spinal cord tissues [44–46]. Mitogenic agents such as EGF and FGF promote spinal cord NSC proliferation *in vitro* and *in vivo*, and EGF promotes migration from the central canal [47–49]. VEGF, whose expression level is increased after SCI, activates spinal cord NSC proliferation through VEGFR2 and EGFR signal pathways [37]. Spinal cord extracts of rats after SCI promote embryonic rat NSC proliferation *in vitro* through elevating Notch1 and Hes1 expression, and Notch pathway activation might be one component in the injury niche of the spinal cord that promotes NSC self-renewal [50].

### 3.2. Spinal Cord NSC Migration and Differentiation

Migration of the activated NSCs away from the central canal towards the lesion site can be detected by 3 days after spinal cord injury [10, 51]. The migrating cells change their morphologies and lose expression of FoxJ1, Sox2, and Sox3 [10]. Reports show that the recruited NSCs at lesion sites mainly differentiate into astrocytes and, to a less degree, oligodendrocytes, but into no neurons after injury [10]. Yet controversy still exists in the field. Using Nestin-Cre-ERT transgenic mice as a tracing model, Ren et al. and Zukor et al. indicated that ependymal cell-derived progenies contribute minimally to the protective scar-forming astrocytes [52, 53].

## 4. Cellular Strategies for SCI Repair

**Figure 2:**
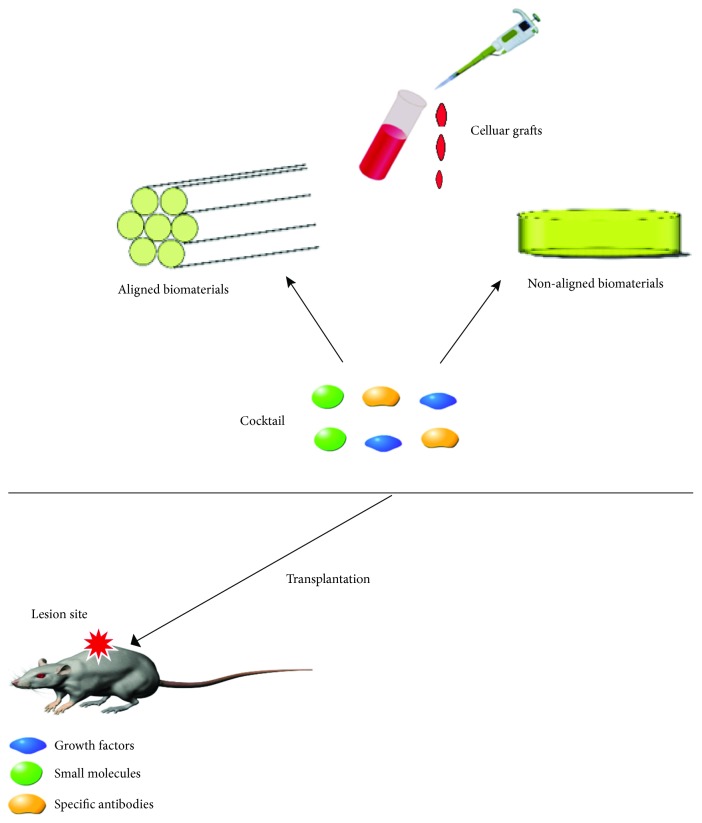
Combined cellular strategies for SCI repair.

Spinal cord injury is a neurodegenerative disease that results in loss of neurons, astrocytes, and oligodendrocytes, leading to physical impairments [54, 55]. In addition, inflammatory reactions, ischemia, and apoptosis cause secondary damage to spinal tissues. Cell therapy, or combined with the administration of growth factors and/or biomaterials, has shown promising potentials for SCI repair [56–61] (Figure 2). Transplantation of derivatives of embryonic cells, induced pluripotent stem cell (iPSC), NSC, or induced NSC (iNSC) has produced regenerative effect and partial recovery from injury [62–64].

Culture of mouse embryonic stem (ES) cells was first reported by Evans and Kaufman in 1981 [65] and human ES cells by Thomson and colleagues in 1998 [66]. ES cells can be easily propagated *in vitro* and form teratoma when injected *in vivo*; therefore, ES cells need to be differentiated into neural lineage precursors prior to transplantation. Studies have shown that neurally differentiated cells derived from mouse ES cells, when transplanted into a rat spinal cord 9 days after traumatic injury, could survive and differentiate into astrocytes, oligodendrocytes, and neurons and migrate as far as 8 mm away from the lesion boundary. Engraftment also improves the hind limb functions of the injured rats [67]. Transplantation of human ES-derived oligodendrocyte progenitor cells (OPCs) promotes remyelination and restores locomotor performance after SCI [68].

iPSCs are generated by a reprogramming process through overexpression of transcription factors such as Sox2, Klf4, Oct4, and c-Myc (SKOM) as reported by Takahashi and Yamanaka in 2006 [69]. Generation of iPSCs can also be realized via viral transduction, mRNA transfection, and/or small molecules [69–75]. iPSCs can be easily expanded to a large scale in culture and also of an autologous origin, thus circumventing most of the immune recognition-associated problems. However, a manufacturing scale-up process may affect the differentiation ability of iPSCs and thus the purity of desired derivatives accordingly; enrichment by applying immune-magnetic beads may be used to address this issue in some contexts. Researchers have grafted human iPSC-derived neurospheres into the injured mouse spinal cords, and the grafts can form synapses and improve locomotor recovery [76, 77].

ES and iPSCs are pluripotent and can be differentiated into many lineages of cells, and thus have remarkable potentials to be used in a wide spectrum of conditions; however, the clinical application of ES cells is complicated by ethical problems; NSCs derived from embryos have similar issues in some countries, albeit to a less degree. Use of iPSC-differentiated NSCs may circumvent these ethical issues. Researchers have examined embryonic NSCs in repair of SCI in animal models. Neurons derived from transplanted NSCs extracted from embryonic forebrains restore disrupted neuronal circuitry in mouse SCI models; nevertheless, another study shows that NSCs from the E14 rat cerebral cortex or the adult rat subventricular zone are restricted to a glial lineage when engrafted into the normal or lesioned spinal cord [78, 79]. The seemingly difference between these two studies may be due to the fact that the former study transplanted embryonic NSCs together with valproic acid (VPA), which may promote neuronal differentiation from NSCs [79]. Okubo and colleagues have reported that iPSC-derived neural progenitor cells, together with gamma-secretase inhibitors, promote functional recovery in the subacute and chronic phases of SCI [80, 81], and they are proposing an initiative to conduct a first-in-human clinical trial using hiPSC-NPCs to treat chronic SCI patients [82].

## 5. Potential of Endogenous Spinal Cord NSCs for SCI Repair

Even though exogenous cell transplant may promote recovery after SCI, the carcinogenic risk, the invasive nature, and complications associated with transplantation procedures pose some challenges in the field. Furthermore, it is difficult to fully control the fate of the transplants [83]. Ependymal cells have been shown as the endogenous spinal cord NSCs. Some animals such as tailed amphibians exhibit powerful endogenous neurogenic capacity and are able to almost fully repair their damaged spinal cords and functionality after SCI [84–87]. Turtles spontaneously reconnect their severed spinal cords, leading in some cases to substantial recovery [88]. It has attracted a lot of interest with the idea that endogenous spinal cord NSCs might contribute to functional recovery. An in-depth understanding of the molecular mechanisms underlying the regeneration-permissive niches in these organisms may lend critical knowledge to help promote endogenous neurogenesis of the mammalian spinal cord after injury [89]. Also, it is reasonable to predict that, given the complicated nature of SCI, an individual approach targeting a single molecule/pathway may not be sufficient to offer a panacea, and tailoring specific combinations of therapies would provide a better outcome [90].

Ependymal cells differentiate into astrocytes to form scar tissues, and about half of the scar-associated astrocytes are derived from ependymal cells. The astrocytes migrate towards the core of the scar and produce laminin that is helpful for axon growth [91]. The GFAP^+^ astrocytes derived from the resident astrocytes are localized in the brim of the scar and secrete chondroitin sulfate proteoglycans (CSPG) which are inhibitory to axon growth [92, 93]. Eliminating ependymal cell-derived astrocytes or total reactive astrocytes in the scar tissue may enhance immune cell infiltration and lead to enlarged lesion volume, increased neuronal death, and aggravation of the functional outcome [94, 95]. Blocking the generation of ependymal cell progenies results in 79% animals failing to form compact scar tissues, secondary enlargement of lesions, and further axonal loss [96]. In addition, the recent studies report that astrocyte scar aids in axon growth, and RNA sequencing reveals that astrocytes and nonastrocytic cells in SCI lesions express multiple axon growth supporting molecules [97]. More interestingly, in fresh water turtles, the activated ependymal cells contributed to the generation of cycling cells that are an important part of the reconstructive bridging scaffold permissive for axon regrowth after SCI [98].

Isolation of ependymal cells from SCI rats and transplantation of these cells into severe contusion models lead to long-distance migration from the transplant bolus to the neurofilament-labeled axons in and around the lesion zone [99]. Retrovirus-mediated overexpression of the Neurogenin2 and Mash1, with growth factor treatment, enhances the production and maturation of new neurons and oligodendrocytes, when directly injected into the injured spinal cord [100]. Spinal cord NSCs also possess a property of plasticity. For example, adult spinal cord-derived stem cells that normally do not generate neurons after injury can differentiate into interneurons if injected into the adult hippocampus [101, 102]. These studies indicate that ependymal cells have a potential to repair SCI, given sufficient conditions to manipulate the intrinsic properties of these cells and/or the surrounding microenvironment [103]. Furthermore, enhanced physical activity promotes the proliferation and differentiation of endogenous ependymal cells, indicating a key role of exercise on SCI recovery [104, 105]. Siegenthaler et al.'s work showed that voluntary exercise attenuates age-related reparative deficits following contusion SCI and the recovery rate of locomotor functions in injured aged rats is comparable to that of injured young rats without excise [106].

## 6. Discussion

There are 3 types of dividing cells in an intact spinal cord, NG2^+^/Olig2^+^ oligodendrocyte progenitors, GFAP^+^/CX30^+^/Sox9^+^ astrocytes, and FoxJ1^+^ ependymal cells, which constitute around 80%, <5%, and <5%, respectively, of the dividing cells. Among these 3 types of cells, only ependymal cell-derived neurospheres are multipotent and can generate neurons, astrocytes, and oligodendrocytes *in vitro*. *In vivo*, ependymal cells are activated after injury and mostly differentiate into astrocytes and oligodendrocytes but few neurons. The limited ability to differentiate into neurons may be partly due to the high expression of Notch1 and Hes1 in the niche after injury, which could be one of the components that inhibit neuronal differentiation [107]. A better understanding of the inhibitory microenvironment will help to find means to unleash the neuronal specification potential, both of the endogenous ependymal cells and of the incoming NSC transplants [108, 109]. In addition, how the NSC transplants interact with the endogenous ependymal cells is not very clear and warrants a further study.

Previously, researchers have tried to transplant NSCs into the injured spinal cord immediately after SCI. However, this may not be the optimal time window for cellular intervention. Expression of inflammatory factors is increased in 6-12 hours after SCI and remains elevated in the following 4 days [110]. Astrocytes release various immune chemicals, such as CSPG, which is beneficial for acute SCI recovery but may be detrimental with chronic exposure, and MCP-1, which plays potent roles in the recruitment of macrophages and monocytes. The infiltrated neutrophils and macrophages can secrete myeloperoxidase, MMP-9, TNF-*α*, TGF-*β*, IL-1*α*, IL-1*β*, IL-6, IL-10, iNOS, Arg-1, etc. [111–113]. The inflammatory responses are the major cause of secondary tissue degeneration, namely, secondary SCI. In addition, neurotrophic factors, CNTF for example, which promotes differentiation into astrocytes, are increased after injury. However, factors that promote neuronal and oligodendrocytic genesis, such as NT3 and BDNF, remain at a low level. This contrasting expression of factors that block neurogenesis vs. enhance neurogenesis may be, at least in part, a reason for nonneuronal generation of endogenous NSCs after injury. It is possible that the optimal time for cellular intervention may not be the acute phase after injury. The secretion of inflammatory factors would last for 1 week, and the vascular reconstruction that is beneficial for neurogenesis occurs during 7-14 days [114]. Therefore, some researchers proposed that the optimal time window for cell transplantation might be 7-14 days postinjury [115].

The number of endogenous spinal cord NSCs is small in the central canal, despite their potential for SCI repair. A sufficient number of spinal cord NSCs may be essential to achieve certain reparative effects. To promote proliferation of endogenous NSCs, researchers have attempted to inject VEGF into spinal cords [37] or by electrical stimulation [116]. To promote the differentiation of endogenous NSCs into neurons, researchers have employed linearly organized biomaterials together with drugs such as cetuximab and taxol to repair the injured tissue [117]. However, more work is required to achieve a desirable level of activation and neuronal differentiation of endogenous spinal cord NSCs following SCI.

Furthermore, promoting both neurogenesis and oligogenesis is important for cell therapies for SCI repair. Some molecules or drugs, such as erythropoietin and cetuximab, have been reported to promote differentiation into neurons or oligodendrocytes [118–123]. Repression of the immune system in a balanced manner would also be conducive to recovery. For example, chondroitinase ABC (ChABC) delivery increases the digestion of CSPG and shifts the macrophage towards a M2 phenotype [124]; a single injection of rapamycin, a blocker of the mTOR pathway, reduces macrophage/neutrophil infiltration and inhibits astrocyte activation, leading to increased neuronal survival and axonogenesis towards the lesion site [125]. Finding an optimal interventional strategy, possibly with a combinatory approach, to promote neurogenesis and oligogenesis and eventually reconstruct the damaged neuronal circuitry and functionality, is the goal in the field for SCI repair. Manipulating the intrinsic properties of ependymal cells at the central canal and turning the inhibitory niche at injury site to a permissive one, together with the exogenous application of NSC/neural precursor grafts and modulatory molecules/drugs, may be tailored to suit the complex conditions of individual patients with SCI in the future.
